# Specific TLR4 Blocking Effect of a Novel 3,4-Dihydropyrimidinone Derivative

**DOI:** 10.3389/fphar.2020.624059

**Published:** 2021-02-01

**Authors:** Mingqian Zhou, Yiqi Wang, Xiaoying Lin, Jieping Wan, Chengping Wen

**Affiliations:** ^1^ College of Basic Medical Sciences, Zhejiang Chinese Medical University, Hangzhou, China; ^2^ College of Pharmaceutical Science, Zhejiang Chinese Medical University, Hangzhou, China; ^3^ College of Chemistry and Chemical Engineering, Jiangxi Normal University, Nanchang, China

**Keywords:** 3, 4-dihydropyrimidinones, DHPMs, TLR4, antagonist, inhibition

## Abstract

**Background:** Toll-like receptor 4 (TLR4) initiates both innate and adaptive immune responses, which plays an important protective role in self-defense mechanisms. Excessive or inappropriate TLR4 activation causes the development of many autoimmune diseases. Dihydropyrimidinone derivatives are medicinally important molecules with diverse pharmacological activities, including anti-inflammatory activity. The present study focused on novel synthesized 3,4-dihydropyrimidinone derivatives and evaluated their inhibitory effects on TLR4.

**Methods:** A series of 3,4-dihydropyrimidinone derivatives were recently synthesized and evaluated for their TLR4 inhibition activities and cytotoxic on HEK-Blue^TM^ hTLR4 cells with the help of QUANTI-Blue assay and MTS assay. Selected compound 3 was analyzed for its molecular docking with TLR4 by using Autodock vina 1.1.2. Its effect on the TLR4 pathway related cytokines was also evaluated in THP-1 cells and human peripheral blood mononuclear cells by using real-time PCR, ELISA and western blot.

**Results:** Five compounds were synthesized and characterized for effectiveness based on 3,4-dihydropyrimidinone. Compound 3 was found to be the potent hybrid among the synthesized compounds, with high TLR4 inhibition activities and low cytotoxic activities against HEK-Blue^TM^ hTLR4 cells. Molecular docking analysis showed that two hydrogen bonds between compound 3 and residues Asp209(TLR4) and Asp99(MD-2) mainly contribute to the TLR4 inhibition. In addition, compound 3 suppressed LPS-induced of the mRNA expression of TLR4, IP-10, TNF-α, IL-6, IL-12A, and IL-12B, the protein expression of pIRF3 and pNFκB and the secretion of IP-10, TNF-α in THP-1 cell line. Compound 3 also inhibited LPS-induced expression of TNF-α, IL-6, and IL-1β but increased IP-10 at mRNA levels in human peripheral blood mononuclear cells.

**Conclusion:** Our study reveals compound 3, a novel 3,4-dihydropyrimidinone derivative, is a potential TLR4 antagonist, which opens up new research avenues for the development of promising therapeutic agents for inflammatory and autoimmune diseases.

## Introduction

The TLR family is a class of pattern recognition receptors (PRRs) of mammalian species, which can recognize many pathogen-associated molecular patterns (PAMPs) and is important in both innate and adaptive immune responses (Kawasaki and Kawai, 2014). TLR4 was the first discovered member of the TLR family and its most important PAMP is lipopolysaccharide (LPS), the main component of the cell wall of Gram-negative bacteria. Activating of TLR4 by LPS after the infection of Gram-negative bacteria will result in the release of inflammatory cytokines and type I interferon, which plays a protective role in self-defense mechanism. However, over activation of TLR4 can lead to serious inflammatory reaction and cause damage to the body, such as in sepsis. Recently, many endogenous molecules such as heat shock proteins (HSPs), Tenascin-C, S100 proteins, and high mobility group box 1 (HMGB1) have also been found to be ligands of TLR4 (Tsan and Gao, 2004; Kiyeko et al., 2016; Bhattacharyya and Varga, 2018). Excessive activations of TLR4 by these endogenous ligands were related to many diseases such as arthritis, systemic sclerosis, and atherosclerotic (Erridge, 2010; Kiyeko et al., 2016; Bhattacharyya and Varga, 2018). Therefore, TLR4 becomes a new target for treating cancer, autoimmune diseases, fibrosis, brain ischemia and neuralgia etc. So far, some TLR4 specific antagonists (e.g., eritoran, (+)- naloxone, resatorvi) have been found (Takashima et al., 2009; Matsunaga et al., 2011; Lewis et al., 2012; Shirey et al., 2013; Wang et al., 2016; Dickinson and Wondrak, 2018; Ono et al., 2020). However, due to toxicity, selectivity and other reasons, there are no drugs for clinical use. Therefore, there is still a great need to discover new type of TLR4 antagonist.

Dihydropyrimidinones (DHPMs) are well-known heterocyclic scaffolds of remarkable pharmacological interest with abundant biological relevance including antiviral, antitumor, antibacterial and anti-inflammatory activities (Wan and Pan, 2012; Matos et al., 2018). Recently, dihydropyrimidinones have been found to be potential anti-inflammatory agents. Some dihydropyrimidinone derivatives have been identified as promising anti-inflammatory drugs by carrageenan-induced paw edema assays in rats and mice (Mokale et al., 2010). The anti-inflammatory action of dihydropyrimidinones involves inhibiting the expression of cytokines and chemical mediators, including TNF-α, inter-leukin, prostaglandin, iNOS, hyaluronidase and COX-2, most of which are regulated by TLR4 (Matos et al., 2018). Accordingly, it has great potential to find new TLR4 antagonist in dihydropyrimidinones.

Herein, we synthesized five compounds based on 3,4-dihydropyrimidinone and evaluated their TLR4 signaling inhibitory effect *in vitro*. We identified and functionally characterized one of the compounds with low toxicity and relatively high TLR4 antagonistic activity, which suggests it can be used as a potent lead compound in the development of new TLR4 receptor antagonists.

## Materials and Methods

### Materials

Lipopolysaccharides from *Escherichia coli* O55:B5 (Cat# L2880) was purchased from Sigma-Aldrich Co., LLC. (MO, United States). Normocin (Cat# ant-nr-1), Phorbol myristate acetate (PMA) (Cat# tlrl-pma) and QUANTI-Blue (Cat# rep-qb2) were purchased from InvivoGen (San Diego, CA, United States). CellTiter 96® AQueous One Solution Cell Proliferation Assay (MTS) was purchased from Promega Corporation (WI, United States). The human TNF-α ELISA kit was purchased from Thermo Fisher Scientific (CA, United States). The human IP-10 ELISA kit was obtained from R&D SYSTEMS (MN, United States). Antibodies against phospho-IRF3 (Ser396), IRF3, phospho-NFκB p65 (Ser536), NFκB p65 and β-actin were purchased from Cell Signaling Technology (MA, United States). Goat Anti-Mouse IgG peroxidase conjugate and Goat Anti-Rabbit IgG peroxidase conjugate was obtained from Jackson ImmunoResearch (PA, United States). All other reagents were obtained from commercial sources and used directly.

### Chemical Synthesis of New Dihydropyrimidinones

Compounds 1 and 2 were synthesized following literature procedure reported by Pan et al. (Wan and Pan, 2009). Compounds 3 and 4 were synthesized following literature procedure reported by Wan et al. (Wan et al., 2014). Compound 5 was synthesized following a modified procedure based on literature process reported by Pan et al. (Wan and Pan, 2009). p-Trifluoromethyl benzaldehyde (0.3 mmol), ethyl acetoacetate (0.3 mmol) and thiourea (0.35 mmol) were mixed in 2 ml DMF in a vessel, TMSCl (0.6 mmol) was added and the mixture was stirred at 85°C for 12 h. After cooling down to room temperature, 5 ml H_2_O was added to the vessel and the mixture was extracted with ethyl acetate (3 × 10 ml). The combined organic layers were dried overnight with anhydrous Na_2_SO_4_. The product was purified by silica gel chromatography with elution of mixed petroleum ether and ethyl acetate (VPET: VEA = 3:1).

### Cell Culture

HEK-Blue™ hTLR4 cells (HEK293 reporter cells engineered to express human TLR4) were kindly gifted by Prof. Thomas C. Mitchell (University of Louisville), which were designed for studying the stimulation of human TLR4 (hTLR4) by monitoring the activation of NF-κB. The levels of the activation of NF-κB could be easily determined by the quantification of secreted embryonic alkaline phosphatase (SEAP). SEAP activity was assessed by using the QUANTI-Blue assay. THP-1 cells, the human monocytic cell line, were purchased from American Type Culture Collection (ATCC) (Manassas, VA, United States). All cells were cultured in Dulbecco’s Modified Eagle’s Medium (DMEM) containing 10% heat-inactive fetal bovine serum (FBS) and 1% penicillin-streptomycin at 37°C in a humidified atmosphere of 5% CO_2_. Phorbol 12-myristate 13-acetate (PMA) was used to induce the differentiation of THP-1 cells to macrophages (Park et al., 2007). Normocin was additionally added into medium at a concentration of 100 μg/ml in combination with Penicillin/Streptomycin to prevent cell lines from mycoplasma, bacterial and fungal contaminations.

### Evaluation of Toll-Like Receptor 4 Inhibition in HEK-Blue^TM^ hTLR4 Cells

Stimulation of TLR4 in HEK-Blue™ hTLR4 cells was evaluated by testing SEAP secretion using QUANTI-Blue assay. Cells were cultured evenly in 96-well plate at a density of 5 × 10^4^ cells/well for 2 h. 1 ng/ml LPS was used to stimulate TLR4 and different concentrations of new synthetic DHPMs (1.1–100 mg/L) were combined treated with LPS to observe the activity of TLR4 inhibition. Equal volume of medium was set as blank. After 18 h of drug treatment, the plate was centrifuged at 2000 rpm for 3 min to pellet cell debris. 60 μl of supernatant was transferred into a new 96 well plat-bottom plate, and 140 μl QUANTI-Blue regent was mixed into it. The mixture was incubated at 37°C for 15 min. The SEAP activity was detected by reading the OD value at 620 nm with a microplate reader. The values are normalized to the LPS treated group, which was set as 1.

### Cell Viability Assay (MTS)

After 18 h of drug treatment, the 96-well plate with HEK-Blue™ hTLR4 cells was centrifuged at 2000 rpm for 3 min to pellet cells. 20 μl MTS was added into 100 μl cell culture media. The mixture was incubated at 37°C for 2 h. The cell viability was detected by reading the OD value at 490 nm with a microplate reader.

### Molecular Docking

A molecular docking study was performed to investigate the binding mechanism between compound 3 and the human toll-like receptor 4 (TLR4) and MD-2 complex using Autodock vina 1.1.2 (Trott and Olson, 2010). The three-dimensional (3D) structure of the human TLR4 (PDB ID: 3FXI) was downloaded from Protein Data Bank (http://www.rcsb.org/pdb/home/home.do). The 3D structure of the compound 3 was drawn by ChemBioDraw Ultra 14.0 and ChemBio3D Ultra 14.0 software. The AutoDockTools 1.5.6 package (Sanner, 1999; Morris et al., 2009) was employed to generate the docking input files. The search grid of the TLR4-MD-2 complex was identified as center_x: 9.261, center_y: 0.905, and center_z: 20.315 with dimensions size_x: 30, size_y: 30, and size_z: 30. The value of exhaustiveness was set to 20. For Vina docking, the default parameters were used if it was not mentioned. The best-scoring pose as judged by the Vina docking score was chosen and visually analyzed using PyMoL 2.3.0 and Ligplot 2.2 software.

### Real-Time PCR

Total RNA was extracted from cells treated by the drugs for 6 h by using ultra-pure total RNA extraction kit (centrifugal column type) (BioTeke, China) and reverse transcribed into cDNA by using the PrimeScript™ RT Master Mix (Perfect Real Time) (Takara Bio, Dalian, China). To examine the mRNA expression levels, cDNA was amplified using Light Cycler 96 (Roche, United States) with SYBR® Premix Ex Taq™ II (Tli RNaseH Plus) (Takara Bio, Dalian, China) and GAPDH was used as loading control. The amplification reaction program was performed as follows: step 1: preincubation (95°C, 120 s); step 2: 3 step amplification (40 cycles of 95°C for 5 s, 60°C for 10 s and 72°C for 10 s); step 3: melting (95°C for 60 s, 55°C for 30 s and 95°C for 30 s). The mRNA levels were expressed as relative quantitation, which were calculated by applying the 2^−ΔΔCt^ method. Primers were designed and synthesized by Generay Biotech (Shanghai, China), and the sequences are shown in Table 1.

**TABLE 1:** The sequences of primers used in this study.

TLR4	Forward	5′-AGA​CCT​GTC​CCT​GAA​CCC​TAT-3′
	Reverse	5′-CGA​TGG​ACT​TCT​AAA​CCA​GCC​A-3′
IP-10	Forward	5′-GTG​GCA​TTC​AAG​GAG​TAC​CTC-3′
	Reverse	5′-TGA​TGG​CCT​TCG​ATT​CTG​GAT​T-3′
TNF-α	Forward	5′-CCT​CTC​TCT​AAT​CAG​CCC​TCT​G-3′
	Reverse	5′-GAG​GAC​CTG​GGA​GTA​GAT​GAG-3′
IL-6	Forward	5′-ACT​CAC​CTC​TTC​AGA​ACG​AAT​TG-3′
	Reverse	5′-CCA​TCT​TTG​GAA​GGT​TCA​GGT​TG-3′
IL-12A	Forward	5′-CCT​TGC​ACT​TCT​GAA​GAG​ATT​GA-3′
	Reverse	5′-ACA​GGG​CCA​TCA​TAA​AAG​AGG​T-3′
IL-12B	Forward	5′-ACC​CTG​ACC​ATC​CAA​GTC​AAA-3′
	Reverse	5′-TTG​GCC​TCG​CAT​CTT​AGA​AAG-3′
IL-1β	Forward	5′-AGC​TAC​GAA​TCT​CCG​ACC​AC-3′
	Reverse	5′-CGT​TAT​CCC​ATG​TGT​CGA​AGA​A-3′
GAPDH	Forward	5′-ACA​ACT​TTG​GTA​TCG​TGG​AAG​G-3′
	Reverse	5′-GCC​ATC​ACG​CCA​CAG​TTT​C-3′

### ELISA

THP-1 cells were cultured evenly in 96-well plate at a density of 5 × 10^4^ cells/well and stimulated with PMA (5 ng/ml) for 48 h. Cells were treated with LPS (100 ng/ml) and different concentrations of compound 3, or with an equal volume of medium. After 18 h of drug treatment, the cell supernatants were harvested and stored at −80°C. Afterward, concentrations of TNF-α and IP-10 in the supernatants were detected using ELISA kit according to its instruction. The absorbance was read at 450 and 570 nm using microplate reader (Thermo Fisher Scientific, United States).

### Western Blot

THP-1 cells were cultured evenly in 6-well plate at a density of 1 × 10^6^ cells/well and stimulated with PMA (5 ng/ml) for 48 h. Cells were treated with LPS (100 ng/ml) and different concentrations of compound 3, or with an equal volume of medium. After 24 h of drug treatment, the cells were washed and lyzed in RIPA lysis buffer for 30 min on ice. The lysate was collected and centrifuged at 12,000 rpm/min for 20 min at 4°C. The supernatant was collected and stored at −80°C. Protein concentration was quantified, and protein combined with 5 × loading buffer was denatured in a 95°C metal bath for 10 min. Equal amounts of protein (50 μg) were separated by 10% SDS-PAGE and semi-dry transferred to polyvinylidene difluoride (PVDF) membranes. After blocking, membranes were washed and incubated overnight at 4°C with primary antibodies against phospho-IRF3, IRF3, phospho-NFκB p65, NFκB p65 and β-actin. Then the membranes were washed and incubated with secondary antibodies (anti-rabbit IgG and anti-mouse IgG) for 2 h at room temperature. Finally, the membranes were washed again and incubated with WesternSure ECL Substrate, the protein bands were scanned, and the intensity was quantified using C-DiGit Blot Scanner (LI-COR, Nebraska, United States).

### Peripheral Blood Mononuclear Cells Isolation

Blood collected in EDTA-containing tubes was gently layered to the Ficoll cushions and spun at 1,500 rpm for 15 min without break at room temperature. After centrifugation, the buffy coat layer full of mononuclear cells was carefully removed. The peripheral blood mononuclear cells (PBMCs) were washed in sterile phosphate-buffered saline (PBS) and resuspended in RPMI 1640 (with 100 U penicillin/100 ug/mL streptomycin, Na-Pyruvate, L-glutamine, 10% human AB serum). Seed 1 × 10^6^ cells per well in 6 well plate for drug treatment and Real-time PCR experiment. The study was approved by the ethics committee of Zhejiang Chinese Medical University (project ID: No. 2015zjtcm-016; date of approval: 05 June 2015). The clinical characteristics of human subjects that PBMCs were obtained from were described in Table 2.

**TABLE 2:** Demographic and clinical characteristics of human subjects. Values represent the mean ± standard error of the mean (SEM).

Parameters	Healthy donors
Age (years)	25.17 ± 0.48
Gender (% women)	33.33
Body mass index (kg/m^2^)	21.89 ± 0.55
Comorbidities	No
Medication	No

### Statistical Analysis

All statistical analyses were performed using the SPSS software 24.0 (SPSS Inc. Chicago, IL, United States). Data were presented as mean ± SEM. Differences between groups were evaluated with one-way ANOVA, followed by LSD or Dunnett’s T3. *p*-values less than 0.05 were considered statistically significant.

## Results

### Novel Dihydropyrimidinones Specifically Blocks Toll-Like Receptor 4 with Low Cytotoxicity

**FIGURE 1:**
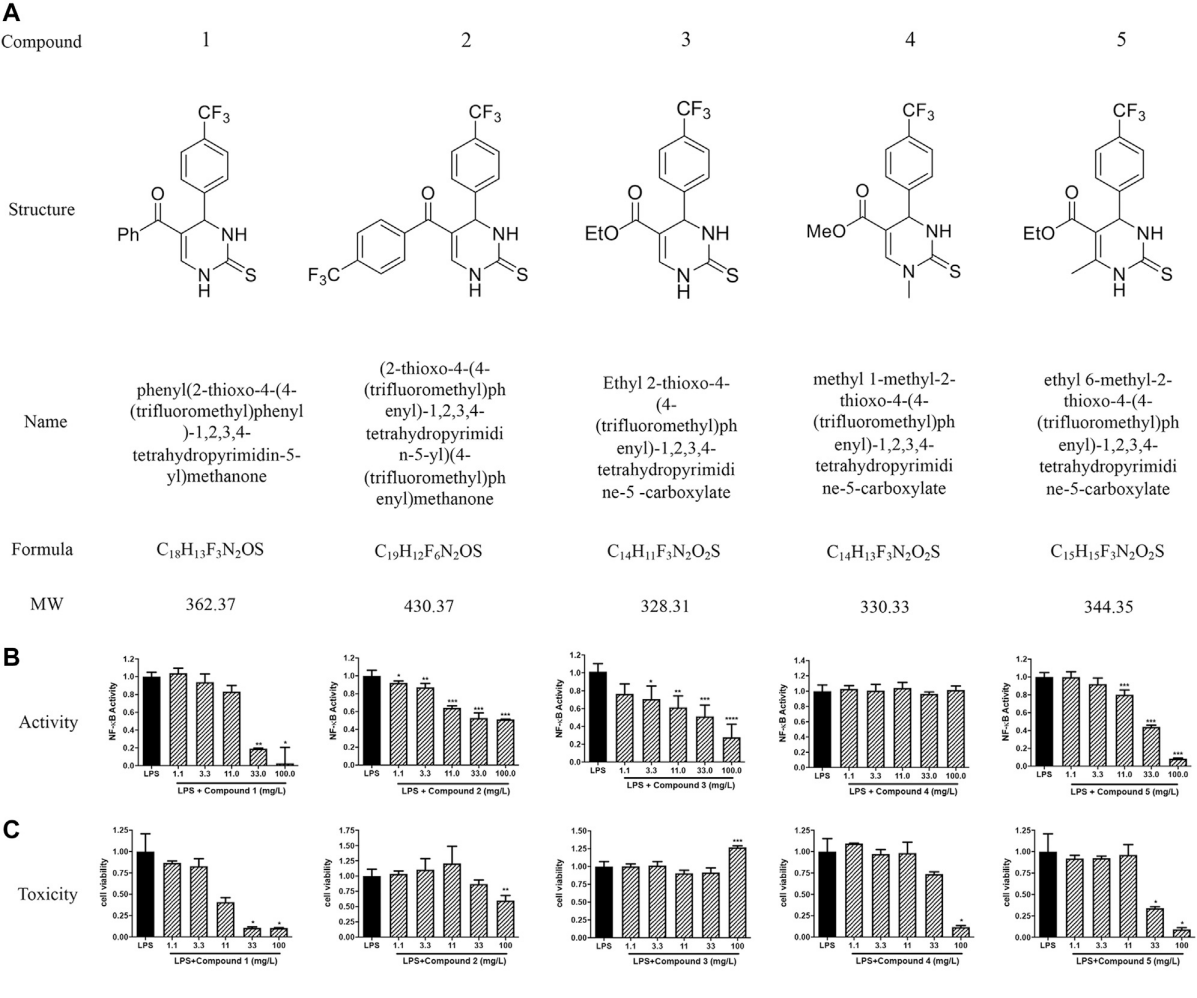
Chemical structure and specific TLR4 inhibitory activity of synthesized DHPMs in HEK-Blue^TM^ hTLR4 cells. **(A)** The chemical structure of DHPMs. **(B)** Effect of DHPMs on LPS-induced TLR4 activation and cytotoxicity. HEK-Blue^TM^ hTLR4 cells were cultured evenly in 96-well plate at a density of 5 × 10^4^ cells/well for 2 h. Cells were stimulated with LPS (1 ng/ml) or TNF-α (10 ng/ml) and different concentrations of new synthesized DHPMs for 18 h. Equal volume of medium was set as blank. Three separate experiments were performed and the mean ± SEM was calculated. *p < 0.05; **p < 0.01; ***p < 0.001; ****p < 0.0001 vs. LPS group or counterparts’ group.

**FIGURE 2:**
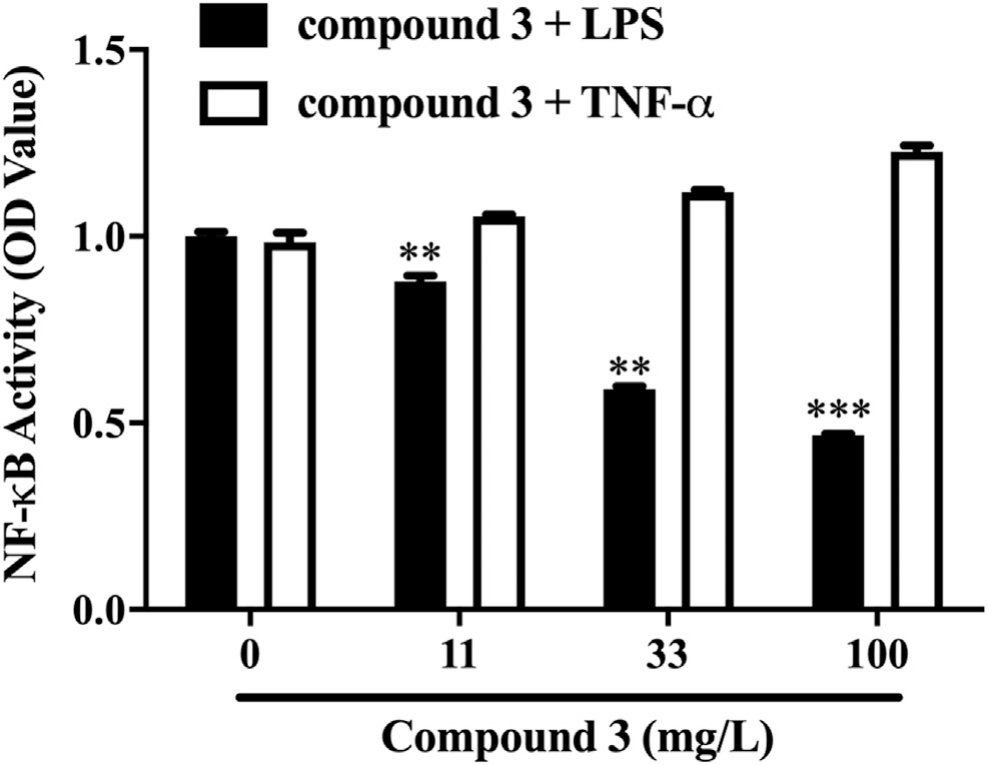
Compound 3 only suppressed LPS induced TLR4 activation but not TNF-α induced TNFR activation. HEK-Blue^TM^ hTLR4 cells were cultured evenly in 96-well plate at a density of 5 × 10^4^ cells/well for 2 h. Cells were stimulated with LPS (1 ng/ml) or TNF-α (10 ng/ml) and different concentrations of compound 3 for 18 h. Equal volume of medium was set as blank. Three separate experiments were performed and the mean ± SEM was calculated. *p < 0.05; **p < 0.01; ***p < 0.001 vs. LPS group or counterparts’ group.

We successfully synthesized five compounds based on 3,4-dihydropyrimidinone and found their specific structure according to our previous work (Wan and Pan, 2009; Wan et al., 2014). The formula and the molecular weight are showed as follows (Figure 1A). ^1^H and ^13^C NMR spectra and other detailed information about compounds are described in Supplementary Material. Among them, only compound 3 showed an outstanding capability of inhibiting the TLR4 pathway and low toxicity on cells, which was assessed by using QUANTI-Blue assay and MTS assay, respectively (Figures 1B,C). Compound 1,2,5, whereas, even showed great effort to inhibiting the TLR4 pathway, was found to be toxic to cells, especially at high doses (Figures 1B,C). Compound 4 had small effects on TLR4 pathway inhibition (Figure 1B). Apart from TLR4, stimulation of other membrane receptors such as TNF-α receptor (TNFR) can trigger NF-κB and JNK pathways which also result in the secretion of SEAP in HEK-Blue™ hTLR4 cells. To determine whether the compound 3 specifically inhibit the activation of TLR4, we use TNF-α to stimulate TNFR at the same time. As shown in Figure 2, compound 3 only suppressed LPS induced TLR4 stimulation but not TNF-α induced TNFR stimulation, which indicates that compound 3 specifically blocks TLR4.

### Compound 3 was Docked Into the Binding Pocket of Toll-Like Receptor 4-MD-2 Complex

**FIGURE 3:**
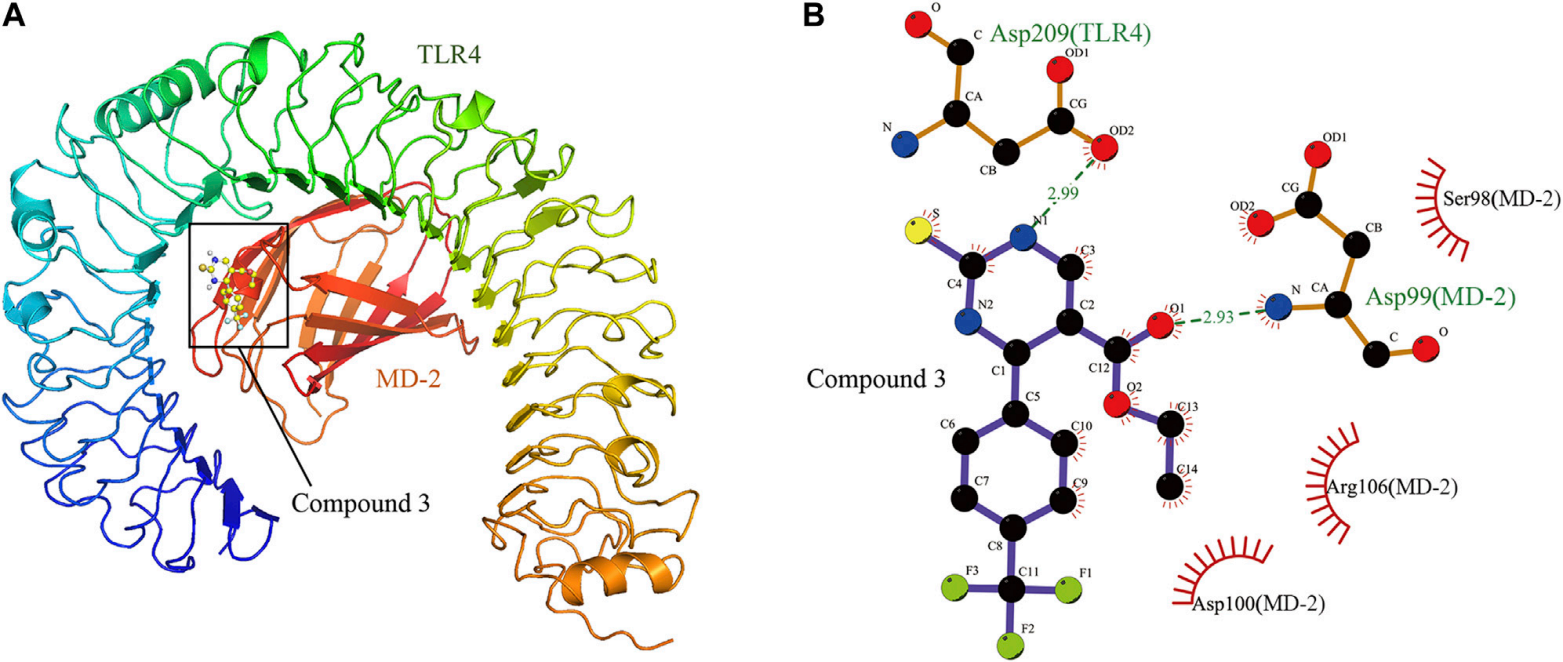
The theoretical binding mode of compound 3 in the binding pocket of TLR4-MD-2 complex. **(A)** Total view. **(B)** Detailed view.

The compound 3 was docked into the binding site of the human TLR4-MD-2 complex and the theoretical binding mode of the compound 3 in the binding pocket of the TLR4-MD-2 complex is illustrated in Figure 3. Compound 3 adopted a compact conformation to bind inside of the pocket of the TLR4-MD-2 complex (Figure 3A). The ester bond of compound 3 was positioned at the hydrophobic pocket, surrounded by the residues Ser98(MD-2), Arg106(MD-2) and Asp100(MD-2) forming a strong hydrophobic binding (Figure 3B). Moreover, two hydrogen bonds were observed between the compound 3 and residues Asp209(TLR4) and Asp99(MD-2), with the bond lengths of 2.99 and 2.93 Å, respectively, which was the main interaction between compound 3 and the TLR4-MD-2 complex. All of these interactions together help compound 3 to anchors into the binding site of the TLR4-MD-2 complex.

### Compound 3 Inhibited the Expression of Toll-Like Receptor 4 and Toll-Like Receptor 4 Related Cytokines in PMA Stimulated THP-1 Cells

**FIGURE 4:**
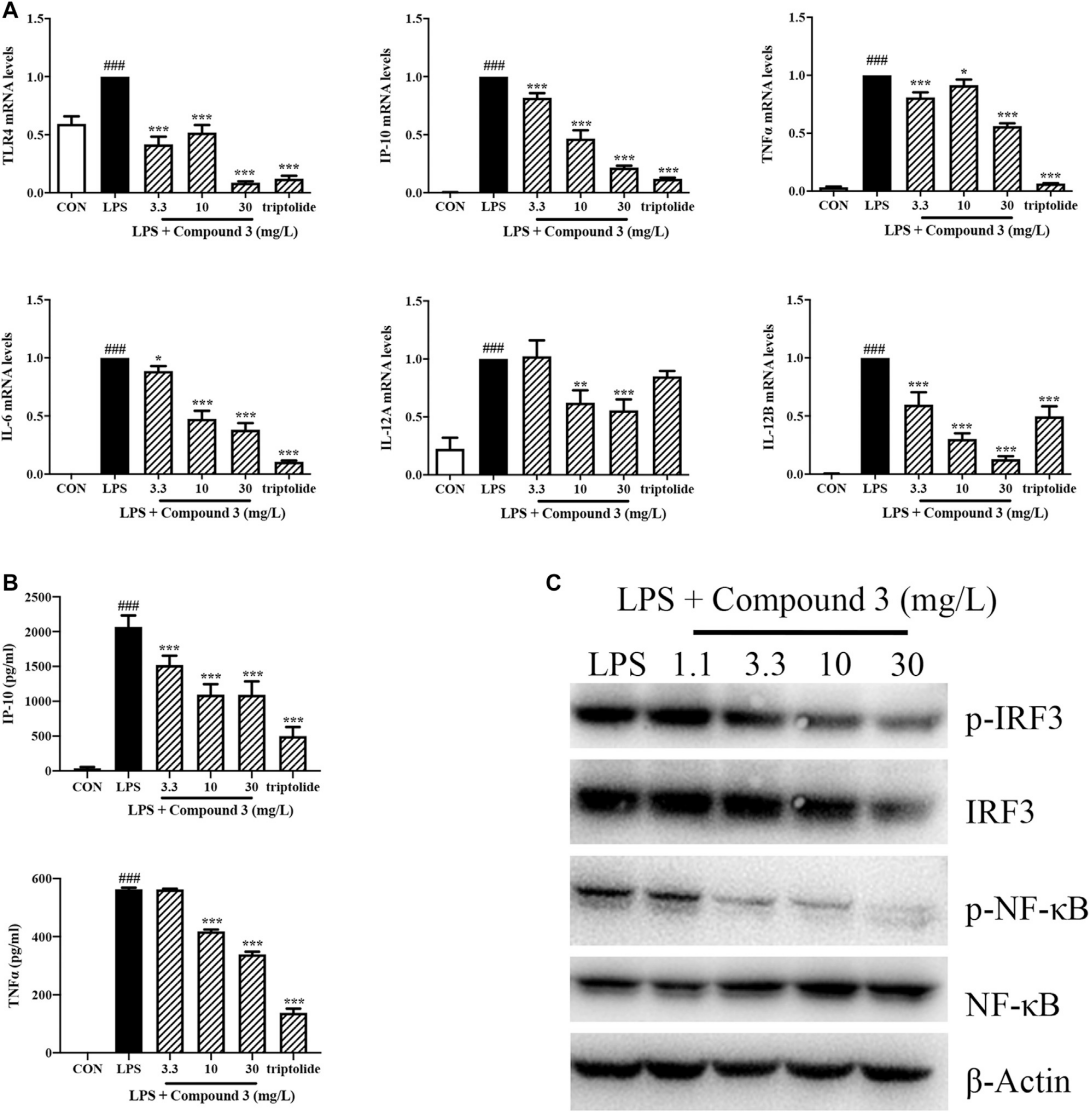
Effect of compound 3 on TLR4 pathway in PMA stimulated THP-1 cells. **(A)** THP-1 cells were cultured evenly and stimulated with PMA (5 ng/ml) for 48 h. Cells were treated with LPS (100 ng/ml) and different concentrations of compound 3, or with an equal volume of medium. RNA was extracted from cells after 6 h of treatment. The mRNA expression was detected by using Real-Time PCR. **(B)** Cell supernatants were harvested after 18 h of treatment and detected by using ELISA kit. **(C)** Cells were harvested and lysed in RIPA lysis buffer after 24 h of treatment. The protein expression of indicated molecules were determined by western blot. Three separate experiments were performed and the mean ± SEM was calculated. *p < 0.05, **p < 0.01, ***p < 0.001 vs. LPS group. ###*p* < 0.001 vs. CON group.

MyD88 and TRIF pathways are the two downstream branches of TLR4. Activation of the MyD88 pathway induces the expression of a variety of MyD88 dependent inflammatory cytokines such as TNF-α, IL-6, IL-12A, and IL-12B. Activation of the TRIF pathway induces the expression of TRIF dependent cytokines such as IP-10. To understand whether compound 3 inhibits both the MyD88 and TRIF pathways, we treated THP-1 cells with LPS and compound 3. The mRNA levels of TNF-α, IL-6, IP-10, IL-12A, and IL-12B were detected using Real-time PCR and the protein levels of TNF-α and IP-10 were detected by using ELISA. As shown in Figures 4A,B, LPS significantly increased the expression of cytokines in both the MyD88 and TRIF pathways, not only at the transcription level but also at the protein level. 10 mg/L of compound 3 was shown to significantly decrease the mRNA expression levels of TLR4 and the related downstream cytokines (*p* < 0.05) after LPS stimulation. The higher concentration (30 mg/L) of compound 3 decreased more significantly (Figure 4A). As shown in Figure 4B, LPS significantly increased the production of TNF-α and IP-10 in cell supernatant while compound 3 dramatically decreased the LPS induced production of TNF-α and IP-10 in a concentration-dependent manner, which is consistent with Figure 4A. NF-κB and IFN regulatory factor 3 (IRF-3) are key downstream molecules of MyD88 and TRIF pathways. Phosphorylation of NF-κB and IRF-3 are important characteristics of activation of MyD88 and TRIF pathways. The results showed the LPS-induced phosphorylation of NF-κB p65 and IRF-3 dose-dependent decreased after treatment with compound 3 (Figure 4C). These data suggest that compound 3 can affect both the MyD88 and TRIF downstream branches of the TLR4 pathway at both transcription and protein level. Triptolide, a compound which has been confirmed to suppress the expression of TLR4 and its downstream proinflammatory cytokines and chemokines in both MyD88 and TRIF-dependent pathways, was used as a positive control (Premkumar et al., 2010).

### Compound 3 Inhibited the mRNA Expression of Toll-Like Receptor 4 Pathway Related Cytokines in Human Peripheral Blood Mononuclear Cells

**FIGURE 5:**
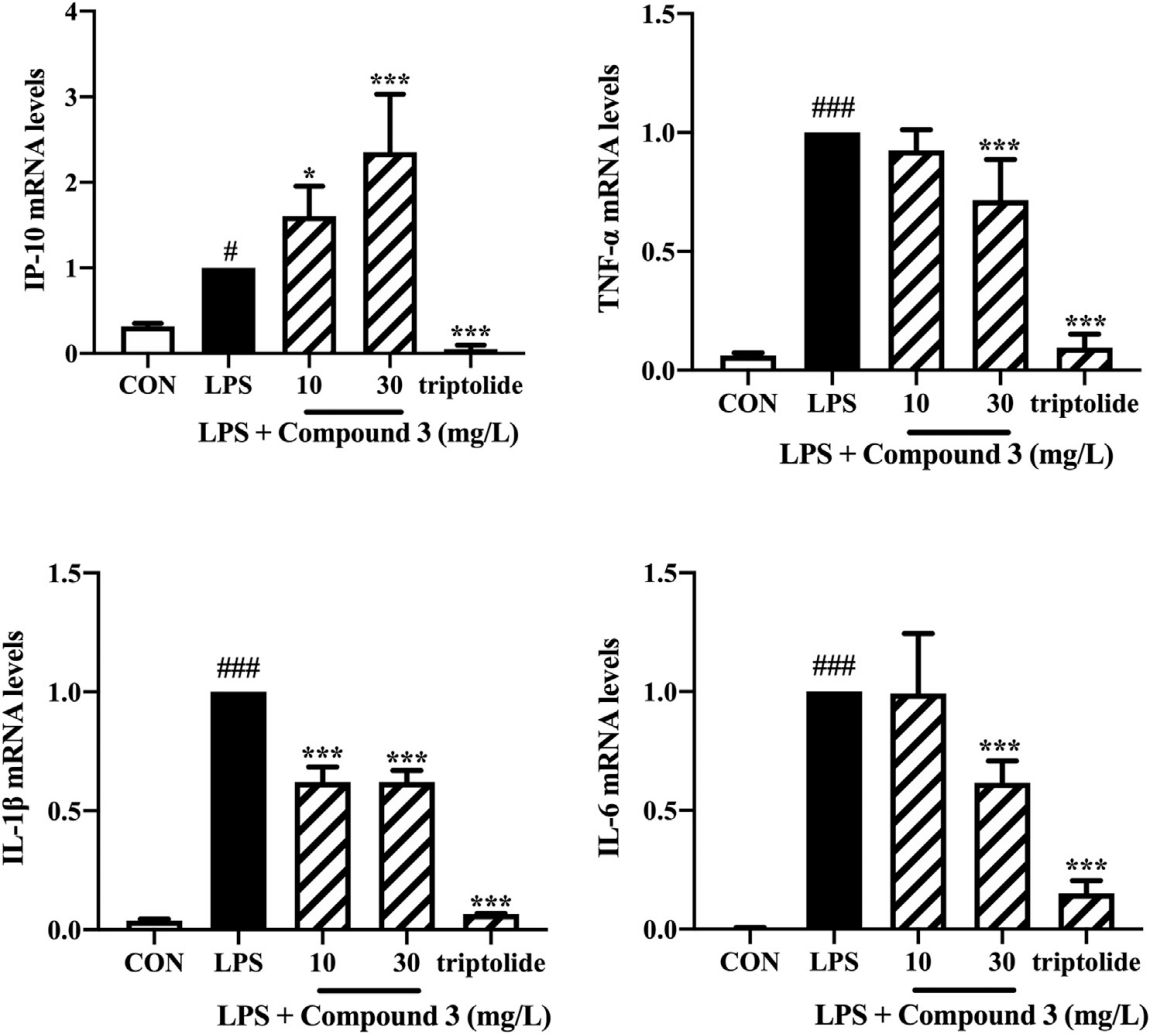
Effect of compound 3 on TLR4 pathway in human PBMCs. PBMCs were cultured evenly in a 6-well plate at a density of 1 × 10^6^ cells/well. Cells were treated with LPS (100 ng/ml) and different concentrations of compound 3, or with an equal volume of medium, for 6 h. The mRNA expression was detected by using RT qPCR. Three separate experiments were performed and the mean ± SEM was calculated. *p < 0.05; ***p < 0.001 vs. LPS group. #*p* < 0.05; ###*p* < 0.001 vs. CON group.

We also detected the mRNA expression of TNF-α, IL-6, IL-1β, IP-10 in PBMCs using Real-time PCR. Triptolide was used to treat PBMCs as a positive control. As shown in Figure 5, 30 mg/L compound 3 can significantly decrease the mRNA expression levels of TNF-α, IL-6, IL-1β after LPS stimulation (*p* < 0.001). Interestingly, comparing with triptolide, compound 3 was shown to have a lighter inhibition, even increase, of IP-10 mRNA expression in human PBMCs.

## Discussion

The main finding of the present study is that the newly synthesized DHPMs can inhibit human TLR4 activation in HEK-Blue™ hTLR4 cells, THP-1 cells and PBMCs, which may become a potent lead compound in the development of new TLR4 receptor antagonists. Toll-like receptor 4 (TLR4) plays an activating role in both innate and adaptive immune responses by specifically recognizing pathogen-associated molecular patterns (PAMPs) and damage-associated molecular patterns (DAMPs), which causes the release of inflammatory cytokines and chemokines and initiates the self-defense mechanisms (Zaffaroni and Peri, 2018). However, excessive or inappropriate TLR4 activation has been directly implicated in many inflammatory and autoimmune diseases, such as ischemia, sepsis, asthma, hepatitis, neuropathic pain, neurodegenerative diseases (Gao et al., 2017; Zaffaroni and Peri, 2018). Unsurprisingly, several TLR4 antagonists have been investigated as potential medicine in an array of diseases (Gao et al., 2017). The most advanced are resatorvi (TAK-242) (Rice et al., 2010) and eritoran (Barochia et al., 2011; Kalil et al., 2011), which have been successful in pre-clinical trial of sepsis but both strategies failed to meet its primary endpoint to reduce the mortality rate in patients with sepsis (Svajger et al., 2013). NI-0101 is the first monoclonal anti-TLR4 antibody entering clinical development, which declared that could block rheumatoid arthritis (RA) synovial fluids-induced pro-inflammatory cytokine production (Hatterer et al., 2016). Disappointingly, the results of phase II clinical trial of NI-0101 makes it stop the pace of progress. The researchers demonstrated that blocking the TLR4 pathway alone does not improve disease parameters, and successful targeting of immune pathways in RA may require broader and/or earlier inhibitory approaches (Monnet et al., 2020). Although great efforts have been made in developing various TLR4 antagonists and many new TLR4 antagonists have emerged in experimental studies (Fang et al., 2014; Foit and Thaxton, 2016), only limited numbers of them have undergone clinical trials, multiple drugs were terminated at different stages during the trials, and none have been approved for clinical uses to date (Fukui et al., 2018; Anwar et al., 2019). Therefore, the identification of new TLR4 inhibitors that can be used as therapeutic agents in clinical still remains an urgent need.

DHPMs are well-known heterocyclic scaffolds with significant pharmacological interest and abundant biological activities including antiviral, antitumor, antibacterial, antihypertensive, antidiabetic and anti-inflammatory activities (Matos et al., 2018). The literature has revealed that the introduction of specific clusters in heterocyclic regions may change their biological activities, and, thus, new compounds are constantly being produced, which exhibit new properties and/or lower cytotoxicity (Prokopcova et al., 2010; Akhaja and Raval, 2011). In this paper we first focus on the effect of DHPMs on TLR4. Herein, we designed and synthesized five novel DHPMs according to our previous work and screened out compound 3 which displayed the best inhibitory activity of TLR4 and low cellular toxicity in HEK-Blue™ hTLR4 cells (Figure 1). Compound 3 has also been shown to specifically inhibit TLR4, by inhibiting only LPS stimulation and not TNF-α stimulation (Figure 2).

As the TLR4-MD-2 dimer is responsible for TLR4 activation (da Silva Correia and Ulevitch, 2002), we performed a molecular docking study to understand the binding mode between compound 3 and the human TLR4-MD-2 complex. The result indicated compound 3 adopted a compact conformation to bind inside of the pocket of the TLR4-MD-2 complex (Figure 3A). The ester bond of compound 3 was positioned at the hydrophobic pocket, surrounded by the residues Ser98(MD-2), Arg106(MD-2) and Asp100(MD-2) forming a strong hydrophobic binding (Figure 3B). Detailed analysis showed that the main interaction between compound 3 and the TLR4-MD-2 complex is the two hydrogen bonds between the compound 3 and residues Asp209(TLR4) and Asp99(MD-2), with the bond lengths of 2.99 and 2.93 Å, respectively (Figure 3B). All of these interactions together help compound 3 to anchors into the binding site of the TLR4-MD-2 complex.

Based on the data obtained with the first screening, compound 3 was further investigated to assess TLR4 pathway inhibitory activity in human monocyte cell line THP-1. The downstream signaling pathway of TLR4 includes two branches: the first is MyD88-dependent pathway, mediated by NF-κB, that produces inflammatory factors such as TNF-α, IL-6, IL-1β and IL-12. Another is MyD88-independent pathway, which is also called the TRIF pathway, mediated by IRF3, that produces interferon-inducible protein-10 (IP-10) and type I IFN (Doyle et al., 2002; Kawai and Akira, 2007; Wang et al., 2019). Most previous studies on the TLR4 inhibitors only focused on TLR4-MyD88 pathway (Palmer et al., 2018; Xu et al., 2018). In this study, we observed the effect of compound 3 on both MyD88 and TRIF pathways. We found compound 3 could suppress the LPS-induced expression of TLR4, IP-10, TNF-α, IL-6, IL-12A and IL-12B at mRNA level *in vitro* (Figure 4A). Consistently, the secretion of LPS-induced inflammatory cytokines TNF-α and immunomodulatory chemokine IP-10 was does-dependently decreased after the treatment of compound 3 (Figure 4B). LPS-induced phosphorylation of NF-κB p65 and IRF-3 also dose-dependently decreased after treatment with compound 3 (Figure 4C), which further confirmed compound 3 inhibits both MyD88 and TRIF-dependent pathway.

We further evaluated the expression of LPS-induced inflammatory cytokines in primary human monocyte, PBMC. PBMCs represent an important cell population for biological evaluation of immunomodulatory compounds because they contain all major monocytes, including those that express TLR4, particularly the dendritic cells and macrophages. Both cell types are rapidly activated upon TLR4 stimulation and produce increased quantities of pro-inflammatory cytokines like TNF-α (Svajger et al., 2013). Consistent with experiment results in THP-1 cells, compound 3 suppresses LPS-induced MyD88-dependent inflammatory cytokines, such as TNF-α, IL-6 and IL-1β in human PBMCs at the mRNA level. Whereas the mRNA expression of the TRIF-dependent chemokine IP-10 was increased (Figure 5). The opposite effect of compound 3 on IP-10 may because PBMCs, as a group of immune cells, contains monocytes, T cells, B cells and some granulocytes which is reasonable to act different to exogenous stimuli than THP-1, an immortalized surrogate monocyte (Riddy et al., 2018; Betsou et al., 2019). The future work will detect the compound 3 effect on various immune cell subsets. In addition, Cheng Qian reported that the regulatory DCs can chemoattract more Th1 cells through IP-10 and then inhibit the proliferation of Th1 cells (Qian et al., 2007), indicating that IP-10 may have a dual manner to maintain immune homeostasis (Liu et al., 2011). Previous study demonstrated that triptolide significantly impaired DC-mediated chemoattraction of neutrophils and T cells by suppressing DC production of CC and CXC chemokines including IP-10 in response to LPS, which induced over-immunosuppressive effect (Liu et al., 2006; Xi et al., 2017). Compare with triptolide, compound 3 has a lighter inhibition, even increase, of IP-10. It suggests that compound 3 may not over inhibit human immune function while playing an anti-inflammatory role.

In summary, the present study has demonstrated for the first time that a novel synthetic 3,4-dihydropyrimidinone derivative, compound 3, was effective in depressing human TLR4 signaling. This compound thus can be used as a lead compound for the development of new human TLR4 antagonists and represents a very promising starting point for pharmacological intervention of TLR4 by DHPMs. The future work will focus on the structural modification of compound 3 and the study of its structure-activity relationship to find more potent compounds. Further experimental animal models will be used to confirm the safety and effectiveness of these compounds in inhibiting TLR4 activation.

## Data Availability Statement

The original contributions presented in the study are included in the article/Supplementary Material, further inquiries can be directed to the corresponding authors.

## Ethics Statement

The studies involving human participants were reviewed and approved by The ethics committee of Zhejiang Chinese Medical University. The patients/participants provided their written informed consent to participate in this study.

## Author Contributions

MZ: performed the research, analyzed data, and wrote the paper; YW: designed the study and edited the article; XL: data collection; JW: developed the idea for the study and synthesize the compounds; CW: conceived of the study and review the article.

## Funding

This work was supported by the National Natural Science Foundation of China (NO. 81603363) and National Key Research and Development Program of China (NO. 2018YFC1705500).

## Conflict of Interest

The authors declare that the research was conducted in the absence of any commercial or financial relationships that could be construed as a potential conflict of interest.

## Abbreviations

TLR4, toll-like receptor 4; DHPMs, dihydropyrimidinones; PRRs, pattern recognition receptors; PAMPs, pathogen-associated molecular patterns; LPS, lipopolysaccharide; HSPs, heat shock proteins; HMGB1, high mobility group box 1; PBMCs, peripheral blood mononuclear cells.
