# Supplementary material for: Specific TLR4 Blocking Effect of a Novel 3,4-Dihydropyrimidinone Derivative
Source: Front Pharmacol. 2021 Feb 1;11:624059. doi: 10.3389/fphar.2020.624059 (PMC7882735; doi:10.3389/fphar.2020.624059)
Supplement: Supplementary file 1 [file datasheet1.docx]

Supporting Information

Experimental details on the synthesis of all DHPMs

**Phenyl(2-thioxo-4-(4-(trifluoromethyl)phenyl)-1,2,3,4-tetrahydropyrimidin-5-yl)methanone** (**1**).^1^ ^1^H NMR (DMSO-*d_6_*, 400 MHz) δ 10.61 (d, 1 H, *J* = 4.8 Hz), 9.88 (s, 1 H), 7.75 (d, 2 H, *J* = 8.0 Hz), 7.58-7.45 (m, 7 H), 6.95 (d, 1 H, J = 5.6 Hz), 5.55 (s, 1 H).

**(2-Thioxo-4-(4-(trifluoromethyl)phenyl)-1,2,3,4-tetrahydropyrimidin-5-yl)(4-(trifluoromethyl)phenyl)methanone** (**2**). ^1^H NMR (400MHz, DMSO-*d_6_*) δ 10.75 (d, 1 H, *J* = 4.4 Hz), 9.96 (s, 1 H), 7.85 (d, 2 H, *J* = 8.0 Hz), 7.79 (d, 2 H, *J* = 8.0 Hz), 7.74 (d, 2 H, *J* = 8.0 Hz), 7.59 (d, 2 H, *J* = 8.0 Hz), 6.99 (d, 1 H, *J* = 8.0 Hz), 5.56 (d, 1 H, *J* = 3.2 Hz); ^13^C NMR (100MHz, DMSO-*d_6_*) δ 191.3, 174.5, 147.5, 142.1, 139.4, 131.6 (q, *J_C-F_* = 31.7 Hz), 129.4, 128.8 (q, *J_C-F_* = 31.5 Hz), 128.1, 126.2 (q, *J_C-F_* = 3.3 Hz), 125.9 (q, *J_C-F_* = 3.7 Hz), 125.7 (q, *J_C-F_* = 270.9 Hz), 112.8, 53.6.

**Ethyl 2-thioxo-4-(4-(trifluoromethyl)phenyl)-1,2,3,4-tetrahydropyrimidine-5 -carboxylate** (**3**). ^1^H NMR (400MHz, DMSO-*d_6_*) δ 10.43 (s, 1 H), 9.62 (s, 1 H), 7.76 (d, 2 H, *J* = 8.0 Hz), 7.46 (dd, 2 H, *J* = 7.2 Hz, 7.6Hz), 7.22 (d, 1 H, *J* = 5.2 Hz), 5.32 (s, 1 H), 4.07 (t, 2 H, *J* = 8.0 Hz), 1.15 (t, 3 H, *J* = 6.8 Hz).

**Methyl 1-methyl-2-thioxo-4-(4-(trifluoromethyl)phenyl)-1,2,3,4-tetrahydropyrimidine-5-carboxylate** (**4**). ^1^H NMR (CDCl_3_, 400 MHz) δ 7.71 (s, 1 H), 7.59 (d, 2 H, *J* = 8.0 Hz), 7.43 (d, 2 H, *J* = 8.4 Hz), 7.31 (s, 1 H), 5.40 (s, 1 H), 3.68 (s, 3 H), 3.56 (s, 3 H); ^13^C NMR (CDCl_3_, 100MHz) δ 177.3, 164.9, 145.8, 137.2, 130.6 (q, *J_C-F_* = 31.8 Hz), 127.2, 126.0 (q, *J_C-F_* = 3.8 Hz), 125.3 (q, *J_C-F_* = 270.7 Hz), 107.0, 54.8, 51.9, 41.7.

**Ethyl 6-methyl-2-thioxo-4-(4-(trifluoromethyl)phenyl)-1,2,3,4-tetrahydropyrimidine-5-carboxylate** (**5**). ^1^H NMR (400MHz, DMSO-*d_6_*) δ 10.47 (s, 1 H), 9.74 (s, 1 H), 7.75 (d, 2 H, *J* = 8.0 Hz), 7.44 (d, 2 H, *J* = 8.0 Hz), 5.27 (s, 1 H), 4.02 (q, 2 H, *J* = 7.2 Hz), 2.32 (s, 3 H), 1.01 (t, 3 H, *J* = 7.2 Hz); ^13^C NMR (100MHz, DMSO-*d_6_*) δ 175.0, 165.4, 148.2, 146.2, 128.7 (q, *J_C-F_* = 31.6 Hz), 127.8, 126.1 (q, *J_C-F_* = 3.5 Hz), 126.0 (q, *J_C-F_* = 270.3 Hz), 100.5, 60.2, 54.2, 17.7, 14.4.

^1^H and ^13^C NMR spectra of compounds

^1^H of compound 1. ^13^C NMR of compound 1 was reported by Pan et al (1).

^1^H and ^13^C NMR of compound 2

^1^H of compound 3. ^13^C NMR of compound 3 was reported by Wan et al (2).

^1^H and ^13^C NMR of compound 4

^1^H and ^13^C NMR of compound 5

References

1. Wan, J.-P.; Pan, Y. Chemo-/regioselective synthesis of 6-unsubstituted dihydropyrimidinones, 1,3-thiazines and chromonesvia novel variants of Biginelli reaction. Chem. Commun. 2009, 2768-2770.

2. Wan, J.-P.; Lin, Y.; Hu, K.; Liu, Y. Secondary amine-initiated three-component synthesis of 3,4-dihydropyrimidinones and thiones involving alkynes, aldehydes and thiourea/urea. Beilstein J. Org. Chem. 2014, 10, 287-292.
